# LMA® Gastro™ Airway for endoscopic retrograde cholangiopancreatography: a retrospective observational analysis

**DOI:** 10.1186/s12871-020-01019-5

**Published:** 2020-05-13

**Authors:** Andre Tran, Venkatesan Thiruvenkatarajan, Medhat Wahba, John Currie, Anand Rajbhoj, Roelof van Wijk, Edward Teo, Mark Lorenzetti, Guy Ludbrook

**Affiliations:** 1 Discipline of Medicine, The University of Adelaide, Adelaide, South Australia Australia; 2 Department of Anaesthesia, The Queen Elizabeth Hospital, 28 Woodville Rd, Adelaide, South Australia 5011 Australia; 3 Department of Gastroenterology, The Queen Elizabeth Hospital, 28 Woodville Rd, Adelaide, South Australia Australia; 4 Discipline of Acute Care Medicine, The University of Adelaide, Adelaide, South Australia Australia

**Keywords:** LMA® GASTRO™ airway, Endoscopic retrograde cholangiopancreatography, Airway management, Endoscopy

## Abstract

**Background:**

Various airway techniques have been employed for endoscopic procedures, with an aim to optimise patient outcomes by improving airway control and preventing hypoxia whilst avoiding the need for intubation. The LMA® Gastro™ Airway, a novel dual channel supraglottic airway technique, has been described as such a device. Its utility alongside sedation with low flow nasal cannula and general anaesthesia (GA) with intubation for endoscopic retrograde cholangiopancreatography (ERCP) procedures was evaluated.

**Methods:**

Details of all the ERCPs performed in our institution from March 2017 to June 2018 were carefully recorded in the patients’ electronic case records. Data on the successful completion of ERCP through LMA® Gastro™ Airway; any difficulty encountered by the gastroenterologists; and adverse events were recorded. Episodes of hypoxia (SpO_2_ < 92%) and haemodynamic parameters were compared across the three groups: LMA® Gastro™ vs. sedation with low flow nasal cannula vs. GA with an endotracheal tube (ETT).

**Results:**

One hundred seventy-seven ERCP procedures were performed during the study period. The LMA® Gastro™ Airway was employed in 64 procedures (36%) on 59 patients. Of these 64 procedures, ERCP was successfully completed with LMA® Gastro™ Airway in 63 (98%) instances, with only one case requiring conversion to an endotracheal tube. This instance followed difficulty in negotiating the endoscope through LMA® Gastro™ Airway. No episodes of hypoxia or hypercapnia were documented in both LMA® Gastro™ and GA with ETT groups. One sedation case with nasal cannula was noted to have hypoxia. Adverse intraoperative events were recognised in 2 cases of LMA® Gastro™: one had minimal blood stained secretions from the oral cavity that resolved with suctioning; the other developed mild laryngospasm which resolved spontaneously within a few minutes.

**Conclusion:**

In patients undergoing ERCP, the LMA® Gastro™ airway demonstrated a high success rate for ERCP completion. Ventilation was well maintained with minimal intraoperative and postoperative adverse events. This technique may have a role in higher risk groups such as high ASA (American Society of Anesthesiologists) status, or those with potential airway difficulties such as high body mass index and those with known or suspected sleep apnoea.

## Core tip

The aim of this retrospective observational analysis was to evaluate the utility of the LMA® Gastro™ Airway as an airway technique for endoscopic retrograde cholangiopancreatography (ERCP) procedures, in order to improve airway control, prevent hypoxia and avoid the need for intubation. Out of 177 ERCP procedures performed during the study period, the LMA® Gastro™ Airway was employed in 64 procedures (36%) on 59 patients. Of these 64 procedures, the LMA® Gastro™ airway demonstrated a high success rate (98%) for ERCP completion, with only one case requiring conversion to an endotracheal tube. Ventilation was well maintained with minimal intraoperative and postoperative adverse events. This technique may have a role in higher risk groups such as high ASA (American Society of Anesthesiologists) status, or those with potential airway difficulties such as high body mass index and those with known or suspected sleep apnoea.

Tran A, Thiruvenkatarajan V. LMA® Gastro™ Airway for endoscopic retrograde cholangiopancreatography: a retrospective observational analysis.

## Background

Endoscopic retrograde cholangiopancreatography (ERCP) is a commonly performed intervention in the management of pancreatico-biliary disorders. The patients presenting for this procedure are usually elderly with significant co-morbidities, and there has been a steady increase in the demand for these procedures. Moderate to deep sedation is a commonly employed technique for ERCPs, with general anaesthesia utilising an endotracheal intubation being reserved for selected cases. Reported rates of hypoxemia during all endoscopic procedures range from 11 to 50% [1–3], and this may be as high as 60% with ERCP [4]. (Definitions of hypoxia vary between the studies.) Sustained hypoxia is a major risk factor for peri procedural cardiac arrhythmias and myocardial ischaemia [5–7]. Some of the anaesthetic challenges of ERCP are the requirement of a semi-prone position, a shared airway, the semi-urgent nature of some of the presentations, and often being required to perform these procedures in a non-operating room environment.

Various airway techniques have been employed for endoscopic procedures, aiming to avoid hypoxia, and obtain better airway control. These include the standard laryngeal mask airway (LMA), gastro-laryngeal tube (GLT), endoscopy mask, a specialised bite block and nasal positive pressure delivery devices [4, 8–15].

The LMA® Gastro™ Airway (Teleflex® Medical, Ireland), is a new device developed specifically for endoscopy procedures. A recent large, prospective observational trial on 292 patients undergoing gastrointestinal endoscopy has shown a 99% success rate for LMA® Gastro™ Airway insertion [16]. Two small case series (< 14 patients) have described their utility for ERCP procedures [17, 18].

The purpose of this observational study was to evaluate the utility of LMA® Gastro™ Airway as an advanced airway technique for ERCP procedures. The specific data assessed on the use of LMA® Gastro™ Airway were: Success rate of completion of ERCPs through LMA® Gastro™ Airway, ventilation and oxygenation parameters, airway related adverse events, and immediate postoperative complications.

## Methods

This study was performed in accordance with the Strengthening the Reporting of Observational Studies in Epidemiology [STROBE] recommendations. This work was considered as a quality assurance study and exempted from ethical approval (Central Adelaide Local Health Network Reference number: Q20190607).

Electronic medical records allowed us to keep track of all patients who underwent ERCP with LMA® Gastro™ Airway from March 2017 to June 2018. This period followed a practice change at our institution when LMA® Gastro™ Airway had just been introduced and some uptake of this device was noted. Selection of the airway technique as moderate to deep sedation assisted by low flow oxygen supplementation, LMA® Gastro™ Airway and general anaesthesia with an endotracheal tube was based on clinical judgement at the discretion of the attending anaesthetist.

Perioperative medical records and discharge summaries were analysed. Data on demographic profile, disease characteristics, preoperative airway assessment, information on airway management including size of LMA® Gastro™ Airway used, any airway manipulations (jaw thrust, chin lift, realigning the device), complications such as bronchospasm, laryngospasm, regurgitation/aspiration of gastric contents, conversion to a different size of the device or endotracheal intubation, data on haemodynamics, oxygenation and ventilation during the procedure and anaesthesia management were collected. Data on the successful completion of ERCP through LMA® Gastro™ Airway and any difficulty encountered by the gastroenterologists were recorded. Any adverse events and adjuvant administered in PACU (post anaesthesia care unit) and reported immediate postoperative pharyngolaryngeal events such as sore throat, dysphagia, dysphonia, and dysarthria were also collected. Hypoxia during ERCP was defined as any documented episode of SpO_2_ < 92% and hypercapnia as an average ETCO2 (end tidal carbon-dioxide) > 45 mmHg. Additionally, the duration of anaesthesia and time spent in PACU were also noted.

An extension to this retrospective study included comparison between three groups: LMA® GASTRO™ vs. sedation with low flow nasal cannula vs. general anaesthesia (GA) with an endotracheal tube (ETT) from March 2017 to June 2018, focusing on demographics, outcomes of hypoxia defined as any incidence of SpO_2_ < 92%, requirement of conversion to endotracheal tube, blood pressure control with vasopressors/inotropes/vagolytics, incidence of adverse intraoperative and postoperative (PACU) events and ERCP failure.

The data were entered in an Excel database and analysed using Microsoft Excel 2017.

## Results

Of the 177 ERCP procedures performed at our institution from 1st March 2017 to the 25th June 2018, LMA® Gastro™™ Airway was employed in a total of 64 procedures (36%), 85 (48%) procedures were done with sedation and 28 (15%) procedures required general anaesthesia with an endotracheal tube. It is likely that the choice of sedation and general anaesthesia with an endotracheal tube would have been based on the clinical profile. Data on the 64 ERCPs utilising the LMA® Gastro™ Airway intervention is presented. Patient demographics, clinical characteristics and the periprocedural data are presented in Tables 1 and 2. Notably, 4 LMA Gastro ERCP cases had a BMI over 40, maximum being 44. All of them tolerated the procedure well. A majority of the cases in this LMA Gastro group were ASA 3 or 4 (59.7%, 37 out of 62).

**Table 1:** Baseline patient clinical characteristics. Results are presented as number (%) or median (range) for continuous data

Characteristics	*n*
Demographics	
Male/Female	28/36
Age (years)	66 (27–91)
Average BMI kg/m^2^	29 (18–44)
Nature of ERCP	
Elective	37
Emergency	26
ASA Status 1/2/3/4	3/22/32/5
Anticipated difficult airway^a^	
Yes	10
No	49
Unknown	5
Relevant comorbidity	
Suspected/Known OSA	10
Chronic Obstructive Pulmonary Disease	1
Gastro-oesophageal reflux disease	24
Bronchial asthma	2
Active/Ex- regular tobacco smoking	21
Hypertension	13
Congestive Cardiac Failure (CCF)	5
Ischaemic Heart Disease (IHD)	9

*‘Unknown’ pertains to mean there was a lack of documentation for that many patients*

**Table 2:** ERCP Procedural characteristics. Total *n* = 64. Results are presented as number or mean (range) for continuous data

Procedural characteristics	*n*
ERCP Position^a^	
Lateral	42
Semi prone	11
Supine	1
LMA^®^ GASTRO™ Airway Size^b^	
3/4/5	20/36/3
Anaesthetic agents and adjuvants	
Propofol infusion + Fentanyl	24
Propofol/Alfentanil infusion	39
Muscle relaxant use	1
Hyoscine butyl bromide	12
Vasopressor use	15
Patient Parameters	
Pre-procedural heart rate	76 (48–115)
Lowest heart rate during ERCP	72 (45–115)
Highest heart rate during ERCP	88 (55–144)
Pre-procedural SpO_2_	97 (94–100)
Lowest SpO_2_ during ERCP	98 (92–100)
Highest SpO_2_ during ERCP	99 (95–100)
Lowest EtCO_2_ during ERCP	41 (31–55)
Highest EtCO_2_ during ERCP	44 (33–60)
Lowest BIS value	41 (31–55)
Highest BIS value	44 (33–60)
Mean Duration of Anaesthesia (in minutes)	57 (30–115)
PACU lowest SpO_2_	97 (92–100)
PACU medications	
Nebulisation	32
Opioid analgesia	11
Anti-emetic usage	10
Time spent in PACU (minutes)	56 (9–225)

a**-** data available in 54 procedures

b**-** size not mentioned in 3, one conversion to endotracheal tube

In the LMA® Gastro™ group, the patients were anaesthetised by 14 different consultant anaesthetists. The 64 ERCPs were performed by two gastroenterologists. One particular consultant provided anaesthesia for 26 ERCPs, with the second most common provider anaesthetising 6 ERCPs. The 2 gastroenterologists contributed 43 (66%) and 21 (33%) cases each.

Out of the 64 ERCPs reviewed, LMA® Gastro™ Airway was used as the primary airway device in 63, and in one instance it was used as a rescue airway intervention for a failed sedation technique. Of the 64 procedures, ERCP was successfully completed with LMA® Gastro™ Airway in 63 (98%) instances, with only one requiring conversion to an endotracheal tube. This instance followed difficulty in negotiating the endoscope through LMA® Gastro™ Airway. There were no documented instances of chin lift, jaw thrust, head and neck manipulations, repositioning the airway, or changing the size of the device. No episodes of hypoxia or hypercapnia were documented. Adverse intraoperative events were recognised in 2 cases. One patient had minimal blood stained secretions in the oral cavity that resolved with suctioning; and the other patient had mild laryngospasm which resolved on its own within a few minutes. Two patients were noted to have adverse events in PACU. Laryngospasm resolving within a few minutes was noted in one, whilst another patient developed significant abdominal pain treated with a proton-pump inhibitor and an anti-emetic. No major airway interventions were noted in PACU.

## Comparative data between sedation with low flow Nasal Cannula vs. LMA® Gastro™ Airway vs. Intubation ERCP cases

The distribution of cases between these three airway approaches represented “selected” populations according to anaesthesiologist discretion taking into account the level of patient complexity, risk of aspiration and desaturation, haemodynamic stability, surgical position, user experience and last but not least, patient preference.

Table 3 demonstrates the selectivity of these populations well. None of the patients in the LMA® Gastro™ group and GA with ETT group experienced intraoperative hypoxia. In the sedation group, one case experienced intraoperative hypoxia.

**Table 3:** Comparative Data between all 177 ERCP cases. Results are presented as number (%) or median (range) for continuous data

Characteristics	LMA® Gastro™	Low Flow Nasal cannula	Endotracheal Tube (ETT)
Number of cases (n)	64	85	28
Demographics			
Male/Female	28/36	37/48	10/18
Age (years)	66 (27–91)	73 (19–95)	78 (18–94)
Average BMI kg/m^2^	29 (18–44)	28 (17–44)	29 (18–77)
Nature of ERCP			
Elective	37	52	8
Emergency	26	35	20
ASA Status 1/2/3/4/5	3/22/32/5/0	17/26/35/7/0	1/3/18/6/1
Airway conversion to ETT	1	1	N/A
Intraoperative SpO_2_ < 92%	0	1	0
Lowest Intraoperative SpO_2_	98 (92–100)	98 (89–100)	98 (92–100)
Vasopressor/inotropic/vagolytic use			
Atropine	1	0	1
Adrenaline	1	0	1
Ephedrine	0	1	1
Metaraminol	15	5	9
Noradrenaline	1	0	3
Mean Duration of Anaesthesia (in minutes)	57	51	71
ERCP Failure	6	2	0
Adverse Intraoperative Events^a^	2	3	0
Adverse 24 h Postoperative (PACU) events^b^	2	1	3

*a – adverse events included broncho/laryngospasm, epistaxis, blood-stained secretions and bradycardia*

*b – adverse events included episodes of laryngospasm, apnoea, minor desaturation (SpO*_*2*_ *< 94%) and abdominal pain*

It is unsurprising a majority of ETT cases were emergency procedures needing rapid sequence intubation, maximum BMI was the greatest at 77 and significantly higher in terms of mean duration of anaesthesia time. A large number of the 28 ETT cases were flagged as extremely high risk procedures pre-operatively relating to aspiration risk and airway difficulty, poor oxygen saturation below 95% at baseline, likely extended duration of anaesthesia and prolonged ventilation or airway protection postoperatively, and one case of severe autism requiring general anaesthesia. Of note in PACU, 1 ETT case had a minor desaturation to 94%, another case required ongoing intubation and extended inotropic support, and another demonstrated multiple apnoeic episodes in recovery.

Conversely, a majority of the low flow cannula cases were ASA 1–2, tolerated well with minimal cases needing blood pressure alterations and showed the lowest mean duration of anaesthesia. Similar to LMA® Gastro™, one case required airway conversion to ETT in the context of apnoeic episodes on nasal specs. There was a high incidence of intraoperative events in the sedation group in the setting of bronchospasm, epistaxis, desaturation and bradycardia HR 30–35. Like the LMA® Gastro™ group, the 2 ERCP failures in the low flow cannula group also related to procedural difficulty.

## Discussion

Our observations confirm that the LMA® Gastro™ Airway can be successfully employed as a primary airway technique for ERCP procedures in some patients. The case that required conversion from LMA® Gastro™ Airway to an endotracheal tube was due to the gastroenterologist being unable to get the gastroscope pass through the endoscope channel of LMA® Gastro™ Airway. This happened to be the third case since this technique was adapted by us, possibly noting a difficulty during the early learning phase.

Gastroenterologists are unlikely to adopt the LMA® Gastro™ Airway for complex endoscopic intervention, unless success is demonstrated in both emergency and elective cases across a diverse group of patients. Our study group had a mixture of low and high risk cases giving rise to anaesthetic as well as procedural challenges. Although formal interviews were not conducted, it was evident that the gastroenterologists were satisfied with the device.

A medicolegal analysis of malpractice claims involving anesthesiologists, has shown that gastrointestinal endoscopy procedures comprised the largest portion of “outside operating suite” malpractice claims in the US [19]. Of these, ERCPs represented the maximum likelihood of payout (91% compared with 37.5% of colonoscopies, and 25% of combined endoscopy/colonoscopy procedures). In view of the morbidity associated with endoscopy interventions, there has been an increased interest recently looking for devices that can facilitate better oxygenation and airway control. General anaesthesia with an endotracheal tube may be considered in some ways a “safe option” in the prone position in terms of having a secured airway and a lower ERCP failure rate [20], and there may be a reduction in some complication rates. However, intubation has drawbacks. In addition to the well-known problems associated with insertion of the tube, managing a paralysed intubated patient in a semi-prone position creates additional challenges. Furthermore, there may be a prolongation of anaesthetic time due to the use of muscle relaxants.

Although the first generation laryngeal mask airways have been used successfully for ERCPs, the absence of a dedicated endoscopic channel and a gastric aspiration port are obvious limitations [8–10]. The GLT is perhaps the most widely evaluated supraglottic airway device for endoscopies [4, 11, 12]. Some of the drawbacks of this device include: loss of position of the device after insertion when turning the patient prone, only one size, and it can be used only in patients over 155 cm tall. The design is unfamiliar to many anaesthetists, and its method of use is slightly different compared to other commonly used supraglottic airways.

Difficulty introducing duodenoscope into the oesophagus may be encountered due to a tight/thick crico-pharyngeus muscle and/or significant anterior cervical osteophytes. This can occur especially in the elderly population, either during sedation without airway adjuncts or even under general anaesthesia with endotracheal intubation. Our gastroenterologists believe that this problem was not encountered during their intubation with the duodenoscope in our patient population. It may be attributed to the alignment of the endoscope channel running parallel to the airway lumen communicating distally with the upper oesophageal sphincter where the endoscope exits. This may indicate another potential benefit using LMA® Gastro™ Airway.

**Fig. 1:**
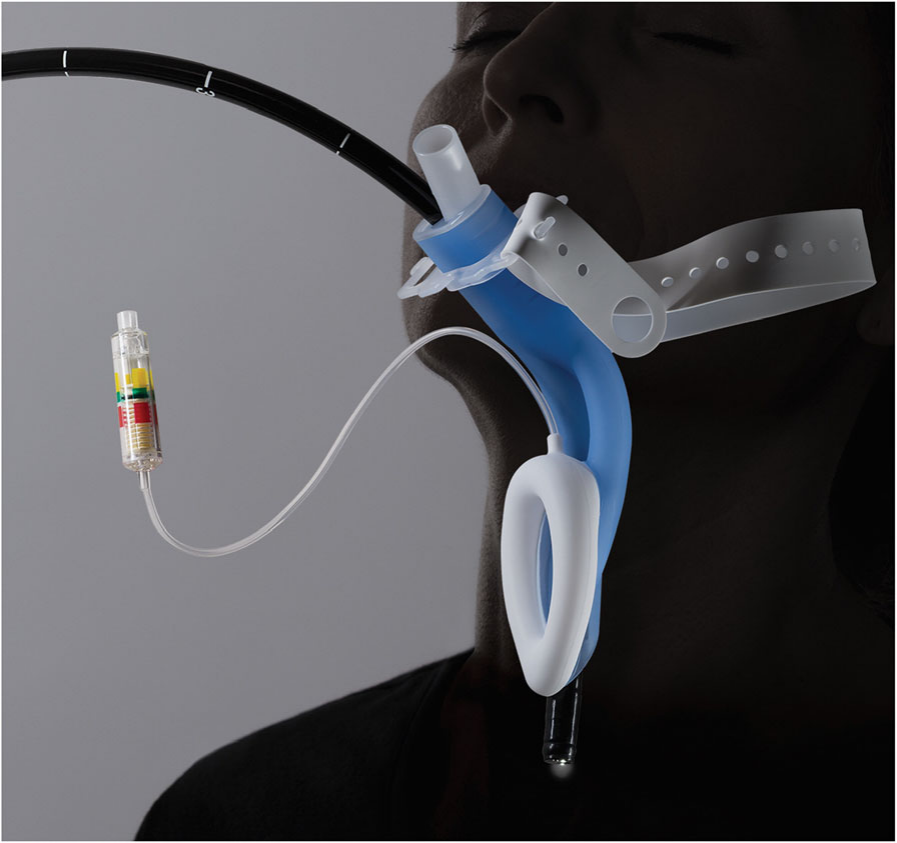
LMA® Gastro™ Airway. Image obtained and adapted from Teleflex, Australia with written permission

The LMA® Gastro™ Airway has dedicated independent channels for both endoscope insertion (16 mm internal diameter) and oxygenation. It also has an integrated bite block, and an adjustable holder to secure the device (Fig. 1). Some of the advantages that are claimed are: improved airway patency, it is available in three sizes: 3, 4 and 5; familiarity and ease of insertion - it is designed similar to other LMAs; insertion possible in lateral or prone position; dynamic flexibility allowing the device to remain in place with head movement; inbuilt cuff pressure monitoring pilot balloon; and allows endoscopes up to 14 mm in size as compared to 13.8 mm with GLT [11].

The 2 cases associated with intraoperative airway events were semi-urgent presentations. Self-resolving mild oropharyngeal bleeding was noted in one. The other emergency case involved an anticipated difficult airway in the context of Down’s syndrome and central obesity (BMI 31). Mild laryngospasm was noted both intraoperatively and in PACU. This was attributed to induction using a volatile anaesthetic in view of needle phobia and the patient’s airway characteristics. Although the procedure was completed with LMA® Gastro™ Airway, the anaesthetic team recommended the use of an endotracheal tube for similar procedures in the future.

Interestingly, LMA® Gastro™ Airway was employed as a rescue technique in one instance where there the SpO_2_ dropped to 86% despite the application of dual nasopharyngeal airways and high flow nasal oxygen therapy. The ease of insertion in a non-supine position and enabling successful ventilation is one of the notable features of this device. Although not formally evaluated, our patients positioned themselves in either lateral or prone position prior to preoxygenation. Unlike other endoscopy airway adjuvants, the LMA® Gastro™ Airway offers reliable CO_2_ monitoring. Oxygenation and ventilation were well maintained in all our cases.

ERCP outcome failure was reported in 5 occasions. While failed cannulation of the bile duct was attributed in three, inability to cannulate ampulla and failed stone extraction were identified in one each. It was evident that the failures were not due to the choice of LMA® Gastro™ Airway as an airway intervention. There is an argument that the endoscope manipulation may be difficult from the extra-oral end of a supraglottic device, rather than a more proximal oropharyngeal entry offered by other airway adjuvants [21]. Nonetheless, the success rate shown in our study diminishes this concern.

It is a contentious issue as to whether non-anaesthesia providers could deliver deep sedation with propofol for a complex intervention such as ERCP [22]. The practice varies globally. Monitoring brain function, some sources have shown that 96% of patients consenting for moderate to deep sedation for endoscopy (including ERCP) were indeed under deep general anaesthesia [23]. The sedation practice (deep propofol based) for endoscopy in Australia is predominantly driven by anaesthetists [24]. A survey on ERCP practice across gastroenterology practitioners in Australia performing the intervention revealed that 97.5% of their cases were assisted by anaesthetists [25]. It has been shown that higher ASA category (> 3) patients would require frequent airway manoeuvres during sedation for ERCPs (1). Hence, LMA Gastro may have a greater role in complex interventions attempted on sicker patients.

### Limitations and strengths

This observational study did not allow for formal matched comparison of efficacy and safety with other conventional airway options such as moderate to deep sedation or other airway adjuvants including GA with ETT and sedation with low flow nasal cannula. Choice of the airway technique was at the discretion of the anaesthetist. Hence, confounding factors in patient selection for the LMA® Gastro™ Airway technique could be a further limitation. Nonetheless, this is the largest series analysing LMA® Gastro™ Airway for ERCPs. Over half of the LMA® Gastro™ cases (37 out of 64) were of the ASA III and IV category and difficult airway was anticipated in 10, implying that the technique was employed on a complex case mix. Future large trials are warranted to analyse the safety and cost implications of this technique in specific population groups such as those with known or suspected sleep apnoea, high BMI and diverse co-morbidities.

## Conclusion

In patients undergoing ERCP, the LMA® Gastro™ Airway demonstrated a high success rate of ERCP completion. Ventilation was well maintained with minimal intraoperative and postoperative adverse events. While the technique may not be required for low risk patients, it may have a role in high risk groups such as high ASA (American Society of Anesthesiologists) status, high body mass index and those with known or suspected sleep apnoea.

## Data Availability

All data generated or analyzed during this study are included in this published article and its supplementary information files. Raw data are available upon reasonable request from the corresponding author.

## Abbreviations

ASA: American society of anesthesiologists
BIS: Bispectral index
BMI: Body mass index
CO_*2*_: Carbon dioxide
ERCP: Endoscopic retrograde cholangiopancreatography
ETCO_*2*_: End tidal carbon dioxide
ETT: Endotracheal tube
GA: General anaesthesia
GLT: Gastro-laryngeal tube
LMA: Laryngeal mask airway
OSA: Obstructive sleep apnoea
PACU: Post anaesthesia care unit
SpO_*2*_: Peripheral oxygen saturation
STROBE: Strengthening the Reporting of Observational Studies in Epidemiology
